# Chronic Intradiploic Organizing Hematoma of the Skull Mimicking Calvarial Tumor Diagnosed Using Zero TE MRI: A Case Report and Review of Literature

**DOI:** 10.3390/medicina57010018

**Published:** 2020-12-28

**Authors:** Hyun Park, In Chul Nam, Hye Jin Baek, Kyeong Hwa Ryu, Eun Cho, Seung Soo Kim, Hyo Jung An

**Affiliations:** 1 Department of Neurosurgery, Gyeongsang National University School of Medicine and Gyeongsang National University Changwon Hospital, 11 Samjeongja-ro, Seongsan-gu, Changwon 51472, Korea; 1coo3004@naver.com (H.P.); chariza@naver.com (S.S.K.); 2 Department of Radiology and Research Institute of Radiology, University of Ulsan College of Medicine, Asan Medical Center, 88 Olympic-ro 43-gil, Songpa-gu, Seoul 05505, Korea; sky_hall@naver.com; 3 Department of Radiology, Gyeongsang National University School of Medicine and Gyeongsang National University Changwon Hospital, 11 Samjeongja-ro, Seongsan-gu, Changwon 51472, Korea; ryukh0329@gmail.com (K.H.R.); sgeisilver@naver.com (E.C.); 4 Department of Radiology, Institute of Health Sciences, Gyeongsang National University School of Medicine, 816-15 Jinju-daero, Jinju 52727, Korea; 5 Department of Pathology, Gyeongsang National University School of Medicine and Gyeongsang National University Changwon Hospital, 11 Samjeongja-ro, Seongsan-gu, Changwon 51472, Korea; ariel2020@naver.com

**Keywords:** hematoma, organizing hematoma, intradiploic space, skull, CT, MRI

## Abstract

Chronic intradiploic organizing hematoma of the skull is a rare lesion that usually presents as a progressively growing mass after head trauma, thus making it difficult to diagnose. To date, only nine cases that have been histopathologically confirmed as organizing hematoma of the skull have been reported in the literature. Herein, we describe a case of a chronic organizing hematoma involving the right parietal bone, presenting as a slowly growing mass in a 54-year-old man. The lesion was also visualized on magnetic resonance imaging (MRI) with a zero echo time sequence. In this case report, we emphasize that chronic intradiploic organizing hematoma should be considered in the differential diagnosis of a palpable scalp mass. We also highlight the importance of meticulous radiological review in the context of appropriate clinical suspicion and the usefulness of the zero TE sequence in evaluating calvarial lesions.

## 1. Introduction

Chronic intradiploic organizing hematoma (CIOH) of the skull is a rare condition that usually presents as a progressively growing scalp mass [1,2]. Although its pathogenesis is controversial, it is believed that it is caused by a benign reparative reaction to traumatic diploic bleeding that is mainly related to minor head trauma [1,2]. To date, only nine cases of CIOH involving the skull have been reported in the literature [1,2,3,4,5,6,7,8,9,10,11]. However, most of these reported cases have shown limited information regarding CIOH, owing to the limited imaging modalities, overall poor quality of representative images, and incongruent description. Herein, we present a case of CIOH in the right parietal bone in a 54-year-old man. The results of computed tomography (CT) and magnetic resonance imaging (MRI) while using an additional zero echo time (ZTE) sequence and histopathological findings are also shown in this report. We also performed a relevant literature research regarding this disease and provided a summary of using the ZTE technique for evaluating calvarial lesions. To the best of our knowledge, this is the first report of CIOH in the skull diagnosed while using the ZTE sequence.

## 2. Case Report 

**Figure 1:**
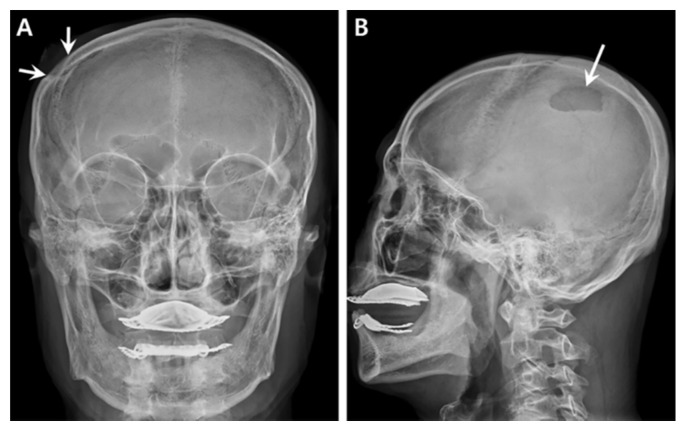
Frontal (**A**) and lateral (**B**) skull radiographs showing a well-circumscribed, oval, osteolytic lesion in the right parietal bone (arrows) with incomplete marginal sclerosis and overlying scalp swelling.

**Figure 2:**
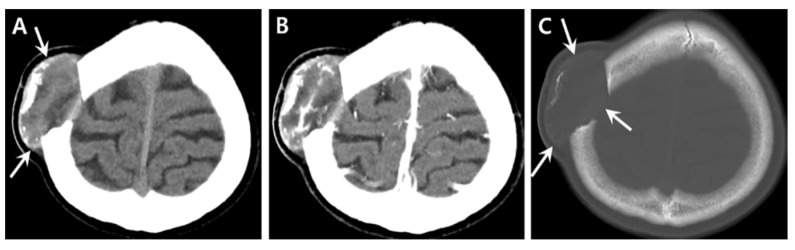
Computed tomography of the brain. (**A**) Soft tissue window setting. The lesion shows inhomogeneous attenuation with marginal capsule and multifocal bone tissues along the outer margin (arrows). (**B**) Contrast enhanced axial image showing multifocal septa-like enhancing components within the lesion. (**C**) Bone window settings. A well-circumscribed, oval, osteolytic lesion is noted in the right parietal bone with multifocal bone tissues in the outer margin, representing the remainder of the outer table (arrows).

**Figure 3:**
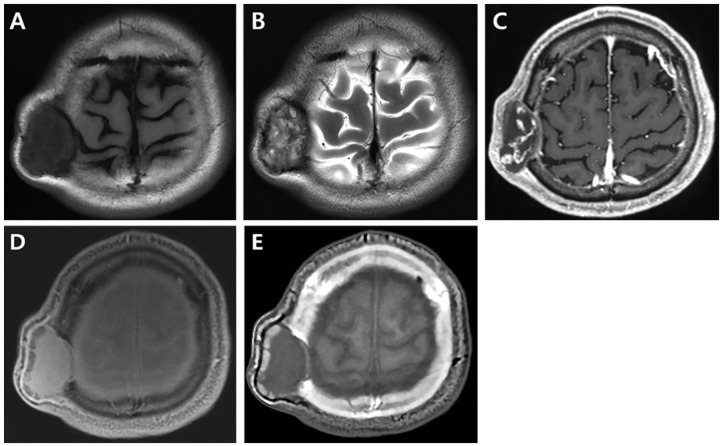
Magnetic resonance imaging of the brain. (**A**) On axial T1-weighted image, the mass demonstrates inhomogeneous hypointensity with dark inner margin. (**B**) On axial T2-weighted image, the expansile intraosseous mass shows heterogeneous hyperintensity with hypointense peripheral rim and internal septa. (**C**) On contrast enhanced axial T1-weighted image, the mass reveals multifocal serpentine enhancing components. There is no combined abnormality in the adjacent brain parenchyma. (**D**,**E**) Axial proton-density (**D**) and computed tomography (CT)-like contrast (**E**) images from zero echo time (ZTE) sequence showing the mass in the right parietal bone with destruction of inner and outer tables and peripheral bone fragments along the outer margin. Findings of ZTE magnetic resonance imaging are similar to those of the CT image with bone window setting (Figure 2C).

A 54-year-old man was referred to our hospital who was complaining of a progressively growing, non-tender scalp mass in the right parietal region for 10 years. He had a history of head trauma due to a car accident in the corresponding region of the palpable mass 25 years ago. Physical examination revealed a hard and fixed mass with a diameter of about 5 cm in the right parietal scalp. There was no sign of combined inflammation. The results of laboratory tests, such as hematological and biochemical tests, were within the normal reference ranges. On the initial neurologic examination, no neurologic deficit was found. Muscle strength and deep tendon reflexes in both the upper and lower extremities were normal. Radiologic studies were requested to evaluate the palpable mass in the right parietal scalp and any intracranial abnormality. On conventional radiography, there was a well-defined, oval osteolytic lesion with incomplete marginal sclerosis in the right parietal bone. Localized irregular scalp swelling was also seen (Figure 1). CT imaging was performed on a dual-layer detector CT unit (IQon Spectral CT, Philips Healthcare, Best, The Netherlands) while using the following acquisition parameters: 120 kVp; 200 mAs; collimation, 64 × 0.625 mm; pitch factor, 0.985; rotation time, 0.33 s; FOV, 250 mm; slice thickness, 3 mm; and, slice increment, 0.4 mm. The CT images revealed a well-circumscribed, osteolytic calvarial mass with inhomogeneous hyperattenuation and septa-like enhancing components in the right parietal bone, measuring 4.8 cm × 4.2 cm in size. This mass was surrounded by a thin hyperdense capsule, like tissue, with the involvement of inner and outer tables of the skull. There were curvilinear deformed bone tissues around the outer margin of the lesion presumably to be a remainder of the outer skull table (Figure 2). In order to characterize this calvarial lesion, brain magnetic resonance imaging (MRI) was performed while using a 3T system (Signa Architect; GE Healthcare, Milwaukee, WI, USA) with a 48-channel head coil. The ZTE skull MRI was also acquired in the axial plane in order to cover the whole brain in 4 min. 52 s while using the following parameters during the same scanning session: TE, 0.016 ms (nominal TE = 0); TR, 2.65 ms; FOV, 240 mm × 240 mm; slice thickness, 2 mm; flip angle, 1°; spokes per segment, 384; matrix size, 288 × 288; image voxel resolution, 0.8 × 0.8 × 1 mm^3^; receiver bandwidth, ±31.25 kHz; and, total number of scans, 3. MRI demonstrated an expansile mass with inhomogeneous signal intensity, showing predominantly hypointense on T1-weighted images and hyperintense on T2-weighted images. The lesion showed multifocal serpentine enhancing foci on contrast enhanced T1-weighted images. However, there was no combined abnormality in adjacent brain parenchyma (Figure 3A–C). On ZTE MR images, the marginal hyperintense foci of the lesion were more clearly depicted than those on the CT images. It was presumed as a residual outer skull table. There was also a conspicuous delineation of thickened dura mater at the inner margin of the lesion on ZTE MR images (Figure 3). 

**Figure 4:**
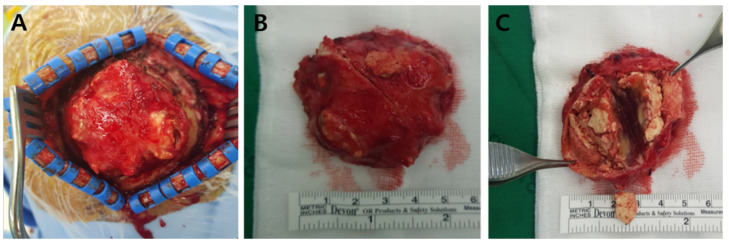
Gross specimen. A 5.0 × 4.5 × 3.0 cm sized, well-defined mass is seen in the right parietal region (**A**). The mass is surrounded by fibrous cap and several ivory-colored bone tissues (**B**). It contains degenerate jelly-like materials and multi-stage hemorrhagic contents (**C**).

**Figure 5:**
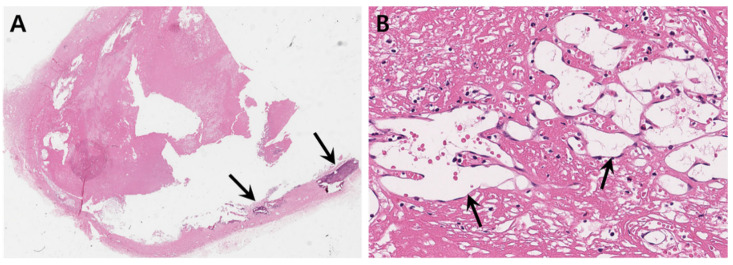
Histopathologic images. (**A**) On low-power field (×10), the lesion reveals a cystically dilated cavitary lesion filled with necrotic fibrinoid material. Hyalinized cystic wall contains dystrophic calcifications (arrows). (**B**) On higher magnification (×200), there are multifocal fibrin thrombi with evidence of recanalization (arrows) and lymphocytic infiltration.

The patient underwent a complete surgical excision of the mass. The neurosurgeon found that outer and inner skull tables were damaged by the mass, and the mass contained multi-stage hemorrhages with jelly-like materials. Several ivory-colored substances in the margin of the lesion detected on CT and MR images. These materials were outer cortical bony structures (Figure 4). The histopathologic findings demonstrated a cystically dilated cavitary lesion that was filled with necrotic fibrinoid materials. The hyalinized cystic wall contained dystrophic calcifications. There were also multifocal fibrin thrombi with evidence of recanalization with multinucleated giant cells, lymphocytes, and foamy histiocytes. These findings were compatible with an organizing hematoma (Figure 5).

## 3. Discussion

CIOH of the skull is a rare condition and the affected sites of the skull include frontal, parietal, and occipital bones. Although the exact pathogenesis is unclear, CIOH is associated with minor head trauma, anticoagulant use, birth trauma, and shunt surgery [1,11]. Most acute hematomas in the skull and scalp usually undergo spontaneous resolution. However, the surrounding connective tissues can encapsulate the hematoma with various stages of differentiation if the diploic hematoma is not resolved [11]. To date, only nine cases of CIOH have been histopathologically confirmed as organizing hematoma of the skull in the literature [1,2,3,4,5,6,7,8,9,10,11] after excluding the hematoma cases finally diagnosed as giant cell reparative granuloma, hemophilic pseudotumor, or subperiosteal hematoma according to the nomenclature that was used by Sato et al. [2]. However, most of these reported cases have demonstrated limited imaging modalities, overall poor quality of the representative images, and incongruent description between the images and figure legends. Therefore, we believe that this case report can provide an accurate summary of CIOH through a meticulous review of the literature (Table 1).

CIOH is often diagnosed on the basis of radiological findings. It is typically presented as a well-circumscribed, expansile osteolytic lesion in the skull with or without surrounding sclerosis with varying attenuations and internal enhancing components on conventional radiography and CT [1,2,3,4,5,7,10,11], as shown in this case. However, Yuasa et al. [3] suggested that CT could not confidently determine whether the lesion is located within the epidural, intraosseous, or subdural space. In contrast to CT, MRI provides more helpful information in order to determine the type of bone lesion, the exact location of the lesion, and the extent of the lesion to adjacent structures, such as brain parenchyma [2]. It provides variable signal intensities, owing to various factors that are related to differentiation and the organization of the hematoma, as follows: age of the blood collection, local tissue oxygenation, form of hemoglobin, breakdown degradation products of hemoglobin, red blood cell integrity, formation and retraction of blood clots, and parameters that are related to the MR machine and technique [10,12,13]. In the present case, imaging findings and possible causes of the lesion that are related to previous head trauma were consistent with previous studies in the literature. The lesion was located in the previous trauma site. It showed inhomogeneous signal intensities on both T1- and T2-weighted images with clearly depicted surrounding capsules. However, the presence of well-defined margins, the absence of perilesional edema, and variable signal intensities suggesting multi-stage hemorrhages are helpful in differentiating CIOH from other space-occupying calvarial lesions, such as tumors and other non-neoplastic lesions, which show solitary osteolytic lesion in the skull vault.

In addition, we applied a novel ZTE sequence for evaluating the skull in the current case. ZTE sequence employs a silent scan algorithm (GE Healthcare, Milwaukee, WI, USA), which uses a nonselective radiofrequency excitation pulse and a three-dimensional (3D) radial center-out k-space trajectory to separate bone from soft tissues and achieve selective bone images [14]. It generates a high-resolution, isotropic CT-like image with positive contrast for the skull by postprocessing [14]. It is well-known that it is unsuitable for depicting cortical bone structure, because of low proton density (approximately 20% of water) and a very short T2 relaxation time (approximately 390 μs at 3T MR system) on conventional MR imaging [15]. Therefore, a CT scan is considered to be the imaging modality of choice for evaluating bone structures with its high spatial resolution, fast acquisition, and high availability in spite of its lower soft tissue resolution [14]. To the best of our knowledge, no study has reported the use of this novel ZTE MRI sequence for evaluating skull masses up to date. In our case, ZTE MR images demonstrated similar findings and image quality of CIOH to CT images. In particular, marginal components proven as the residual outer cortex showed a more conspicuous delineation on ZTE skull MRI than those in other sequences and CT images. Through our initial experience, we believe that ZTE sequence might be useful for evaluating skull lesions in radiosensitive patients, such as children and pregnant women, because it does not generate radiation that is known to be associated with a CT scan.

The differential diagnosis for CIOH should be considered for other calvarial lesions, as follows: (1) benign neoplastic and nonneoplastic lesions; aneurysmal bone cyst, giant cell tumor, giant cell reparative granuloma, fibrous dysplasia, eosinophilic granuloma, intradiploic epidermoid and dermoid cyst, cavernous hemangioma, circumscribed osteomyelitis, tuberculous granulomas, and hemophilic pseudotumor [1,10,16], and (2) malignant neoplasms; metastasis, multiple myeloma, sarcoma, or Langerhans cell histiocytosis [17]. However, in this case, the patient had a history of head trauma, and the lesion presented as a progressively growing mass that was located in the diploic space with expanding to both sides of the cortex. In addition, the lesion had inhomogeneous signal intensities on MR imaging, which suggested multi-stage hemorrhage. Therefore, CIOH was considered to be the most likely radiological diagnosis.

An accurate diagnosis can be established by a histopathological examination, because it is difficult to differentiate CIOH from other diseases, such as giant cell tumors, aneurysmal bone cysts, giant cell repetitive granulomas, cavernous hemangioma, or fibrous dysplasia, which can be radiologically or histologically similar to CIOH [1,11,15]. Complete surgical excision is the treatment of choice for CIOH, and no additional treatment is usually required [1,11]. 

## 4. Conclusions

Herein, we report a case of CIOH in the right parietal bone presenting as a slowly growing mass with clinical, radiological, and histopathological findings. The diagnosis of CIOH can be challenging in clinical practice because of its rarity and nonspecific clinical and radiological features. Therefore, clinical suspicion and awareness are important for accurate diagnosis and management for patients with space-occupying lesions of the skull vault. In addition, a ZTE sequence is useful for evaluating calvarial lesions in a daily practice, because it can easily identify the bony lesions as similar to CT images with its inherent benefit of not generating radiation.

## Figures and Tables

**Table 1:** Reported chronic intradiploic organizing hematoma cases in the skull vault based on histopathological diagnosis in the literature ^†^.

Author/Year	Gender/Age	History of Head Trauma	Clinical Features	Location	Imaging Modality and Findings	Treatment
Yuasa et al., (1992)	M/20 y	Presence (minor trauma)	Incidental scalp swelling	Parietal	Conventional radiography: inner table expansion	Excision and cranioplasty
Sato et al., (1994)	M/20 y	Presence (minor trauma)	Incidental scalp swelling	Parietal	* Note.: overall poor image quality Conventional radiography: hyperostotic change CT: hyperostotic mass like lesion with central low density area MRI: significant enhancing diploic mass with central T2 hyperintensity	Excision and cranioplasty
Uemura et al., (1999)	M/32 y	Presence (minor trauma)	Intermittent headache	Frontal	* Note.: overall poor image quality MRI: expansile intraosseous mass with inhomogeneous T1 signal intensity	Excision
Yücesoy et al., (1999)	M/25 d	Presence (birth trauma)	Scalp swelling	Parietal	* Note.: overall poor image quality CT: isodense calvarial mass with thick osseous layer at the outer border	Excision
Mobbs et al., (2000)	M/3 y	Presence (major trauma)	Non-tender scalp swelling	Parietal	* Note.: incongruent MR images and figures (only T2-weighted and FLAIR images without T1-weighted image) CT: U-shaped bony defect involving the external table and diploe with Irregularly undermined outer edges MRI: inhomogeneous T2 hyperintensity	Curettage
Goel et al., (2000)	M/58 y	Presence (minor trauma)	Swelling and ptosis	Frontal	* Note.: poor image quality of conventional radiography Conventional radiography: expansile osteolytic diploic lesion with sclerotic margin CT: heterogeneously enhancing, expansile intradiploic mass with complete sclerotic borders, extending to the frontoethmoid sinuses	Excision
Batista et al., (2011)	M/45 y	Presence (recurrent traumas due to epilepsy)	Slow-growing painless mass	Parietal and occipital	* Note.: poor image quality of MRI CT: U-shaped bony defect involving the external table and diploe with Irregularly undermined outer edges MRI: expansile intradiploic mass showing heterogeneous T1 hyperintensity and inhomogeneous T2 hypointensity with multifocal peripheral enhancing foci Digital subtraction angiography: Displacement and obliteration of the lower portion of the superior sagittal sinus by the mass on venous phase	Excision
Tokmak et al., (2015)	M/16 y	Presence (minor trauma)	Non-tender scalp swelling	Frontal	* Note.: poor image quality Conventional radiography: round, osteolytic lesion with surrounding sclerosis MRI (T1-weighted image only): homogeneous T1 hyperintensity	Excision
	M/64 y	Presence (minor trauma)	headache and visual disturbance	Frontal	* Note.: poor image quality MRI (T1-weighted image only): dark T1 signal intensity	Curettage

* Note.—^†^ includes only articles available in full-text form in the literature, and we excluded the hematoma cases that were finally diagnosed as giant cell reparative granuloma, hemophilic pseudotumor or subperiosteal hematoma according to the nomenclature used by Sato et al. [2]. In the column of gender/age, M stands for male, d represents days, and y denotes years. CT, computed tomography; MRI, magnetic resonance imaging; and, FLAIR, fluid attenuated inversion recovery.

## Data Availability

The data that support the findings of this study are available from the corresponding author, upon reasonable request.
